# GC Analysis, Anticancer, and Antibacterial Activities of Secondary Bioactive Compounds from Endosymbiotic Bacteria of Pomegranate Aphid and Its Predator and Protector

**DOI:** 10.3390/molecules28104255

**Published:** 2023-05-22

**Authors:** Taghreed Alsufyani, Najwa Al-Otaibi, Noura J. Alotaibi, Nour Houda M’sakni, Eman M. Alghamdi

**Affiliations:** 1 Department of Chemistry, College of Science, Taif University, P.O. Box 11099, Taif 21944, Saudi Arabia; 2 High Altitude Research Centre, Taif University, P.O. Box 11099, Taif 21944, Saudi Arabia; 3 Department of Biology, College of Science, Taif University, P.O. Box 11099, Taif 21944, Saudi Arabia; 4 Laboratory of the Interfaces and Advanced Materials (LIMA), Science Faculty, Monastir University, P.O. Box 05019, Monastir 5019, Tunisia; 5 Chemistry Department, Faculty of Science, King Abdul Aziz University, P.O. Box 80200, Jeddah 21589, Saudi Arabia

**Keywords:** GC-MS, secondary bioactive, anticancer activities, antibacterial activities, Taif-pomegranate, endosymbiotic bacteria, *Aphis punicae*, predator, protector

## Abstract

Bacterial secondary metabolites are a valuable source of various molecules that have antibacterial and anticancer activity. In this study, ten endosymbiotic bacteria of aphids, aphid predators and ants were isolated. Bacterial strains were identified according to the 16S rRNA gene. Ethyl acetate fractions of methanol extract (EA-ME) were prepared from each isolated bacterium and tested for their antibacterial activities using the disk diffusion method. The EA-ME of three bacterial species, *Planococcus* sp., *Klebsiella aerogenes*, *Enterococcus avium*, from the pomegranate aphids *Aphis punicae*, *Chrysoperia carnea*, and *Tapinoma magnum*, respectively, exhibited elevated antibacterial activity against one or several of the five pathogenic bacteria tested. The inhibition zones ranged from 10.00 ± 0.13 to 20.00 ± 1.11 mm, with minimum inhibitory concentration (MIC) values ranging from 0.156 mg/mL to 1.25 mg/mL. The most notable antibacterial activity was found in the EA-ME of *K. aerogenes* against *Klebsiella pneumonia* and *Escherichia coli*, with an MIC value of 0.156 mg/mL. The cytotoxic activity of EA-ME was dependent on the cell line tested. The most significant cytotoxicity effect was observed for extracts of *K. aerogenes* and *E. avium*, at 12.5 µg/mL, against the epithelial cells of lung carcinoma (A549), with a cell reduction of 79.4% and 67.2%, respectively. For the EA-ME of *K. aerogenes* and *Pantoea agglomerans* at 12.5 µg/mL, 69.4% and 67.8% cell reduction were observed against human colon cancer (Hct116), respectively. Gas chromatography–mass spectrometry (GC-MS) analysis of three EA-ME revealed the presence of several bioactive secondary metabolites that have been reported previously to possess antibacterial and anticancer properties. To the best of our knowledge, this is the first study to examine the biological activities of endosymbiotic bacteria in aphids, aphid predators and ants. The promising data presented in this study may pave the way for alternative drugs to overcome the continued emergence of multidrug-resistant bacteria, and find alternative drugs to conventional cancer therapies.

## 1. Introduction

Cancer diseases and the antibiotic resistance phenomenon are two common challenges facing the public health sectors. Various cancers are responsible for millions of deaths each year worldwide Dube, Sakle [1,2]. Traditional therapeutic approaches such as surgery, radiation and chemotherapy are associated with adverse consequences such as anemia, alopecia, hormonal fluctuations, gastrointestinal mucositis and brachial plexopathy [3]. Such deleterious effects have prompted the search for alternative treatment methods [4]. Additionally, antibiotic-resistant bacteria continue to emerge despite the discovery and production of novel antibiotics [5]. Infections with multidrug-resistant bacteria (MDR) usually lead to serious complications, hospitalizations and deaths, and pose challenges to public health authorities around the world. It is therefore urgent to find new antibiotics to address this phenomenon of antibiotic resistance.

Endophytes are considered to be an outstanding source of bioactive natural compounds with anticancer and antimicrobial activities [6]. Endophyte bacteria are less careful than fungal endophytes because of the lower yield concentration of crude extracts in the former [7]. The review by Abdelghani et al. provides information on the raw extracts of bacteria that can be used as antibacterial and anticancer therapies [8]. It is estimated that *Actinomycetes* are responsible for the production of 70% of secondary microbial compounds [9], while Bacilli and other bacteria produce around 7% and 1–2%, respectively [10]. Examples of secondary metabolites used as antibacterial compounds include prodigiosin, methanol-pigmented daptomycin, daptomycin and 3-benzyl-hexahydropyrrolo[1,2-a] pyrazine-1,4-dione from *Serratia marcescens* [11], *Micrococcus* sp. [12], *Streptomyces roseospours* [13] and *Exiguobacterium indicum* [14], respectively. However, anthracyclines, peptides, aureolic acids and antimetabolites produced by *Actinomycetes* [15], and bleomycin from *Streptoalloteichus hindustanus* [16] are also cytotoxic against multiple cancer cells. Isolation of secondary metabolites from natural sources (e.g., insects such as aphids) could be an alternative source of antimicrobial and anticancer chemotherapy that would contribute to reducing problematic infections affecting human health.

Aphids (Hemiptera: Aphididae) are a group of insects of agricultural importance which feed on many plant species. Most aphids establish mutualistic relationships with endosymbiotic bacteria, mainly known as obligate (i.e., primary) and facultative relationships, which are housed in specialized cells called bacteriocytes [17,18]. *Buchnera aphidicola* is a primary model of the obligate symbiont and can be found in almost all aphid species within three clades: *Aphidinae*, *Lachninae*, and *Fordini* [17,19]. *B. aphidicola* plays an essential role in providing amino acids and nutrients lacking in the aphid diet [19]. Nine facultative symbionts, *Serratia symbiotica*, *Hamiltonella defensa*, *Regiella insecticola*, *Rickettsia*, *Rickettsiella*, *PAXS*, *Spiroplasma*, *Wolbachia*, and *Arsenophonus*, have been shown to have positive effects on their hosts such as aphid fitness, immune pathway function and responses to natural enemies (e.g., defense against parasitoid attacks) [20,21] and environmental stress (e.g., adaptation to thermal stress) [22,23,24,25]. However, the biological activities in these endosymbionts have received much less attention. Recent studies have demonstrated antibacterial and antifungal activity in the gall bile tissue of the aphid [26,27], and that the antimicrobial peptide against the pea aphid is a bio-insecticide [28,29]. To the best of our knowledge, no studies have been conducted to investigate the antibacterial and anticancer activities of the endosymbiotic bacteria of aphids, especially in Taif, Saudi Arabia. Only a few attempts have been made to study the morphological identification of aphids in Taif [30], or the control of aphids infesting the Taif rose [31] and Taif pomegranates [32].

It is worth understanding the ecology of aphids to take advantage of the entire environment around the aphid for medical purposes. Aphids are usually associated with ants for the purpose of protection; therefore, the aphid–ant relationship is a typical example of symbiosis [33]. Aphids are also attacked by bioenemies such as lady beetles [34], and lacewing larvae [35].

Here, we hypothesize that the raw extract from the endosymbiont bacteria in aphids and their surrounding insects, whether they are protectors such as ants or bioenemies such as lady beetles and lacewing larvae, exhibit antimicrobial qualities. To test this hypothesis, we first isolated the endosymbiotic bacteria associated with aphids collected from pomegranates, grapes and Taif roses, along with aphid predators (lady beetles and lacewing larvae) and ants, and then we investigated the significant biological activities of the EA-ME extracted from five strains of pathogenic bacteria (*S. aureus*, *S. epidermidis*, *E-coli*, *K. pneumoniae* and *E. cloacae*), and their anticancer activities against two cancer cell lines (adenocarcinoma human alveolar epithelial cells and human colon carcinoma).

## 2. Results

### 2.1. Collection of Insects and Isolation of Insects Endosymbiotic Bacteria

**Figure 1:**
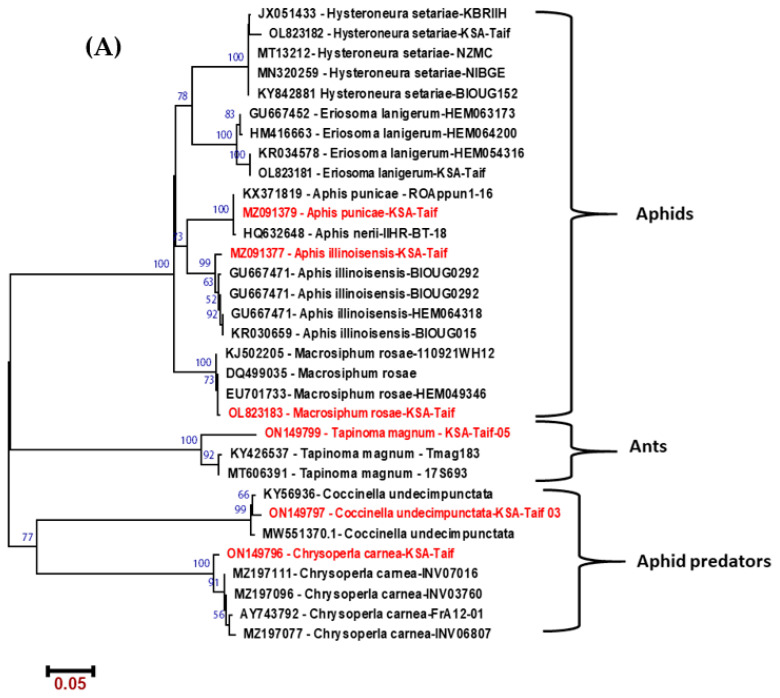

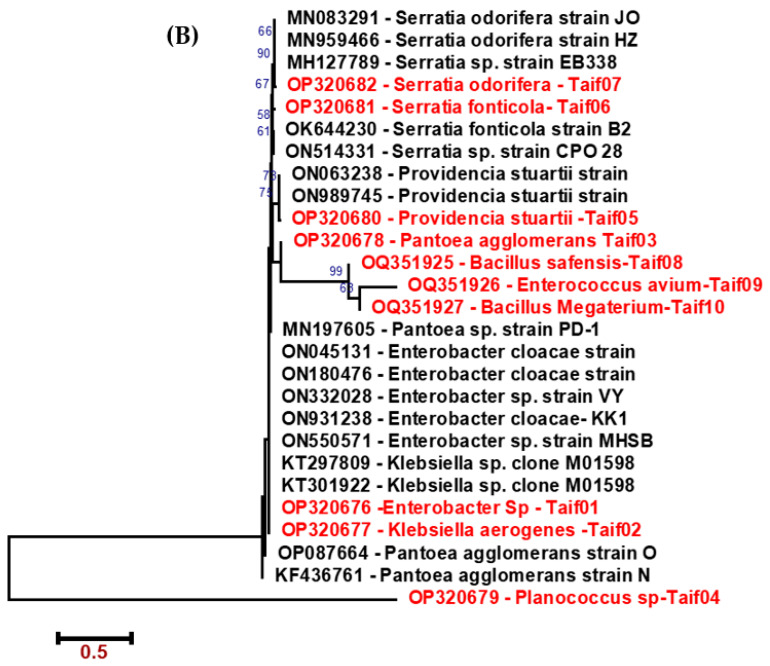
Neighbor-joining phylogenetic tree based on (**A**) mitochondrial COX1 gene of insects used in our study and other similar species from the Genbank database, and (**B**) 16S rRNA gene sequence of the endosymbiotic bacteria isolated from insects and other similar species retrieved from GenBank database. Insects from which bacteria were isolated and 16s rRNA identified bacteria are in red fonts. Numbers above the nodes refer to the bootstrap values generated after 1000 replications.

Three species of aphids were collected and identified molecularly based on the sequence variation in the COI; *A. punicae*, *M. rosae* and *A. illinoisensis* (Figure 1). The identified aphid predators used in this study included *C. carnea*, *C. undecimpunctata*. In addition, one protective species of ant, *T. magnum*. Isolation of endosymbiotic bacteria from insects was attempted over nutrient agar plates. A total of 13 samples of bacteria were isolated and identified through the 16S rRNA gene sequences. The resultant sequences were introduced into the BLAST search tool of the gene bank, resulting in several bacterial genera. As shown in Figure 1, phylogenetic analysis has confirmed the identification of related species grouped into the same clade. Of the identified bacteria, only ten different species were selected to test their antibacterial and antiproliferative activities (Table 5).

### 2.2. Antibacterial Activities of Endosymbiotic Bacteria

No antibacterial activities were recorded for EA-ME after three days of incubation. However, after seven days of incubation, the EA-ME of three endosymbionts, *Planococcus* sp., *K. aerogenes*, and *E. avium*, from *A. punicae*, *C. carnea* and *T. magnum*, respectively, were effective against both Gram-positive and Gram-negative bacteria, with inhibition zones ranging from 10 to 20 mm. The effect of these EA-ME was comparable to the effect of tetracycline against some tested pathogenic bacteria. Extracts of *Enterobacter* sp., *Serratia odorifera*, *Pantoea agglomerans*, and *Bacillus megaterium* had no antimicrobial activities against the tested pathogenic bacteria. The EA-ME of *K. aerogenes* was the most effective against four pathogenic bacteria, *S. aureus*, *S. epidermidis*, *K. pneumoniae*, and *E. coli*, with inhibition zones of 15.00 ± 0.41, 16.00 ± 0.29, 20.00 ± 1.11, and 18.00 ± 0.65 mm, respectively. The EA-ME of *Planococcus* sp. was effective against Gram-positive bacteria; *S. aureus* (17.00 ± 0.75 mm) and *S. epidermidis* (16.00 ± 0.91 mm), with weak antimicrobial activity against *K. pneumoniae* (11.00 ± 0.35 mm).

The EA-ME of the endosymbiont *Bacillus safensis* displayed moderate antibacterial activity against *S. aureus*. According to the MIC values, the EA-ME of *Planococcus* sp. displayed the lowest MIC value (0.3125, *p* ≤ 0.01 mg/mL) against *S. aureus* and *S. epidermidis*. Similarly, the EA-ME of *E.s avium* was effective against *S. epidermidis*, and *K. pneumoniae*, at an MIC value of 0.3125 mg/mL, and the EA-ME of *K. aerogenes* was effective against *S. epidermidis*, at the same MIC value (Table 1). Interestingly, the three EA-MEs displayed low MIC values comparable with those of ampicillin and the second reference antibiotic, tetracycline (Table 1).

### 2.3. Antiproliferative Activities of Endosymbiotic Bacteria

**Figure 2:**
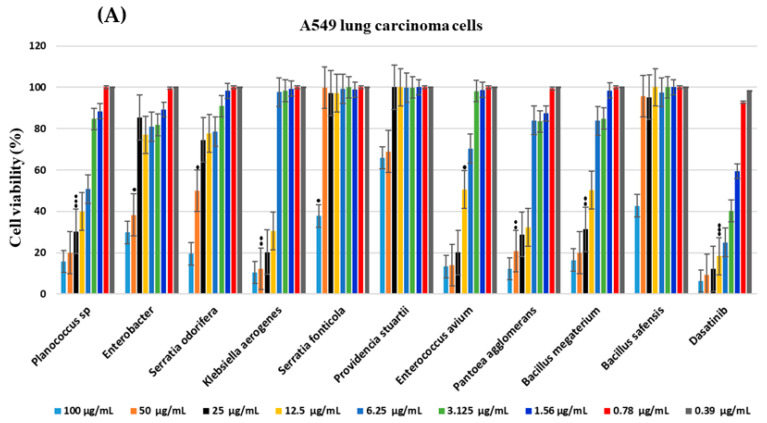

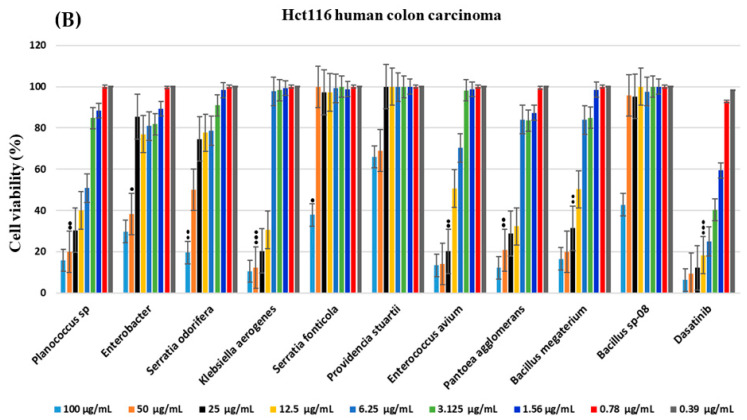
MTT cytotoxic activity assay of insect endosymbiotic bacterial EA-ME tested at different concentrations from 100 µg/mL to 0.39 µg/mL on (**A**) A549 lung carcinoma cells and (**B**) Hct116 human colon carcinoma cells. Bars represent means ± standard deviations (SD) measured for each concentration (*n* = 3). The statistical significance of the IC_50_ values of different extracts and the positive control, dasatinib, were calculated using an unpaired Student’s *t*-test, where (●) *p* < 0.05, (●●) *p* < 0.01 and (●●●) *p* < 0.001.

The cytotoxic activities of ten endosymbiont ethyl acetate fractions of methanolic crude bacterial extracts (EA-ME) were tested against A549 and Hct116 using an MTT assay. In general, the intensity of the cytotoxicity was directly proportional to the concentration of the extracts. A high concentration of EA-ME (100 µg/mL) was effective in reducing the cell growth of A549 and Hct116 cells. The EA-ME of *P. stuartii* displayed the lowest cytotoxic activity against Hct116 (34.1% cell reduction) at a concentration of 100 µg/mL, whereas the same concentration resulted in a 50.1% cell reduction against A549 cells. The EA-ME of *K. aerogenes* displayed the highest cytotoxic activity against A549 cells (92.9% cell reduction) at a concentration of 100 µg/mL. This was followed by the EA-ME of *P. agglomerans* and *Enterococcus avium*, with which cell reduction was 90.9 and 90.0%, respectively. In the case of Hct116 cells, the EA-ME of *K. aerogenes* at a concentration of 100 µg/mL showed an 89.5 % cell reduction, followed by the EA-ME of *P. agglomerans* (87.8% cell reduction) and *E. avium* (86.7% cell reduction) (Figure 2A).

At a low concentration of 12.5 µg/mL, the EA-ME of *K. aerogenes* and *E. avium* displayed the highest cytotoxicity against A549 cells, with a cell reduction of 79.4% and 67.2%, respectively. This was followed by the EA-ME of *P. agglomerans* and *S. odorifera*, with which cell reduction was about 59.2%. The EA-ME of *S. fonticola* and *P. stuartii* had no impact on A549 cells above a concentration of 50 µg/mL. For Hct116 cells (Figure 2B), the EA-ME of *K. aerogenes* and *P. agglomerans* at a concentration of 12.5 µg/mL showed a 69.4% and 67.8% cell reduction, respectively. This was followed by the EA-ME of *Planococcus* sp. and *B. megaterium*, with which cell reduction was 60% and 49.8% at the same concentration, respectively. At a concentration of 6.25 µg/mL, only the EA-ME of *Planococcus* sp. resulted in a 49.8% cell reduction of Hct116 cells. In the case of A549 cells (Figure 2A), the EA-ME of *K. aerogenes* and *E. avium* showed the highest cytotoxicity, with a cell reduction of 59.5% and 45%, respectively. The remaining EA-MEs were ineffective or displayed weak cytotoxicity against both cells at the same concentration. The IC_50_ values were determined for the EA-ME of *Planococcus* sp. (25 ± 8.22) µg/mL against A549 and (48 ± 11.62) µg/mL against Hct116 cells. For the EA-ME of *K. aerogenes*, the IC_50_ values were (67 ± 13.82) µg/mL and (38 ± 9.20) µg/mL for A549 cells and Hct116 cells, respectively. The EA-ME of *E. avium* showed the lowest IC_50_ values of (13 ± 14.13) µg/mL and (22 ± 11.13) µg/mL against A549 cell and Hct116 cells, respectively. The positive control, dasatinib, resulted in a 94.7% (IC_50_: 25 ± 9.1 µg/mL) and 93.7% (IC_50_: 28 ± 13.2 µg/mL) reduction in A549 and Hct116 cells, respectively. The significance of the anticancer activity of the different EA-ME ranged from *p* < 0.001 to <0.05, which is comparable to the positive control, dasatinib.

### 2.4. Chemical Analyses for Endosymbiotic Bacterial EA-ME

The GC-MS results revealed several chemical compounds in each EA-ME; there were 31 compounds in the EA-ME of *Planococcus* sp., 16 compounds in *E. avium*, and 22 compounds in *K. aerogenes.* Among the principal natural compounds identified in the AE-ME of *Planococcus* sp. are folinic acid, benzoic acid, 4-(1,1-dimethylethoxy), 16-octadecadiynoic acid, methyl ester, Z-8-methyl-9-tetradecenoic acid, oleic acid, octadecanoic acid, 9,10-dihydroxy, and methyl ester (Table 2). The EA-ME of *K. aerogenes* also has a number of major compounds: pregnane-3,11,20,21-tetrol, cyclic 20,21-(butyl boronate), acetamide, n-(p-methoxybenzyl), hydrocinnamic acid, o-[(1,2,3,4-tetrahydro-2-naphthyl)methyl], 2,4-dimethylhexanedioic acid, dl-2,6-diaminoheptanedioic acid, and esteoleic acid, (Table 3). In the case of *E. avium*, the most important compounds are 13,16-octadecadiynoic acid, methyl ester, pyrrolo[1,2-a] pyrazine-1,4-dione, hexahydro-3-(2-methylpropyl), pentadecanoic acid, 8,11-octadecadiynoic acid, methyl ester, arachidonic acid, cholest-5-en-3-ol, and cholest-5-en-3-yl (9z)-9-octadecenoate (Table 4). Some of the identified compounds were reported in all three EA-MEs.

The GC/MS of the EA-ME of *E. avium* bacteria isolated from *A. punicae* feeding on pomegranate tree shows the presence of two compounds: mono(2-ethylhexyl) phthalate, which may be detected as a secondary metabolite of di(2-ethylhexyl) phthalate (DEHP), and pregnane-3,11,20,21-tetrol, cyclic 20,21-(butyl boronate) of plant origin (Table 3). Likewise, the GC/MS of the EA-ME from *K. aerogene* bacteria (Table 4), shows the presence of two synthetic contaminants: acetamide, n-(p-methoxybenzyl), and didodecyl phthalate (Table S1).

Phthalic acid esters (PAEs) are lipophilic chemicals widely used as plasticizers and additives to improve the mechanical extensibility and flexibility of various polymers. They have been easily detected in the air, water, soil and sediment not only from synthetic materials but also from living organisms, such as microbes, algae, plants, etc., suggesting they may be biosynthetic in nature [36]. The major PAEs identified from natural sources generally include di-n-butyl phthalate, diethyl phthalate, dimethyl phthalate, di(2-ethylhexyl) phthalate, diisobutyl phthalate, and diisooctyl phthalate. Therefore, di(2-ethylhexyl) phthalate (DEHP) is a plasticizer largely detected in the bio-organism. However, Ref. Bhattacharyya, Dhar [37] shows that the cell-less extract from a cultured *Mycolicibacterium* sp. strain (MBM) grown on DEHP was found to convert DEHP to MEHP and phthalic acid (PA).

Pregnane-3,11,20,21-tetrol, cyclic 20,21-(butyl boronate) was detected in the n-hexane fraction of *Phlomis stewartia* [38] and the methanolic extract of *Epilobium angustifolium* L. [39], and in our study was detected in the EA-ME of *E. avium* bacteria isolate from *A. punicae* feeding on pomegranate tree. Ref. Jansen, Allwood [40] shows that the metabolic interface between the two organisms, the metabolome of the caterpillar, can be modified by the vegetable metabolome via these metabolites. Many studies have focused on several topics of plant–insect interaction at nutrient, molecular, physiological and evolutionary levels [41]. However, a small chemical difference between host plant sources can affect the overall metabolome of specialized herbivores. Significant nutritional changes in herbivorous tissues could cause larger changes in food web structure [40]. The chemical compositions, retention times (RT) and molecular weights are shown in Table 2, Table 3 and Table 4.

## 3. Discussion

The continuous emergence of MDR bacteria and the deleterious effects of traditional cancer treatments have directed researchers to seek alternative approaches. In the past few decades, natural products have made up more than 40% of the drugs approved as anti-microbial or anti-proliferative agents [42]. Bioactive compounds of natural origin have received much attention due to their safety profile, effectiveness, and availability. A huge body of research focuses mainly on medicinal plants, and investigates their anticancer and antibacterial activities against a wide range of pathogenic bacteria and cancer cells [43,44,45]. Another important source of bioactive compounds is endophytic bacteria [6,46,47,48,49,50]. Few studies have investigated the antibacterial and anticancer activities of endosymbiotic bacteria. In these studies, endosymbiotic bacteria such as *bacilli* were isolated from many arthropods [51,52], *Enterobacter* sp. from *Dysidea granulosa* [53] and *Bacillus brevis*, and *Bacillus choshinensis* from the earthworm *Pheretimasp* [54].

In this study, the EA-ME collected after three days of incubation showed neither antibacterial activity nor anticancer activity. Strong biological activities against bacteria andcell lines were obtained from EA-MEs collected after seven days of incubation. After seven days, nutrient depletion in the medium forces bacteria to enter the stationary phase and release secondary metabolites. In several studies, secondary metabolites were obtained after a seven-day bacterial incubation period [5,49,55]. Among the tested EA-ME, *K. aerogenes*’s extract displayed strong antibacterial activities against both Gram-positive and Gram-negative bacteria. Such an antibacterial effect is greater than that of the EA-ME of the *Raoultella ornithinolytica* endophytic bacteria, against *E. coli* and *K. pneumoniae*, for which the MIC values were 0.5 and 0.25 mg/mL, respectively [5]. In another study, the EA-ME of earthworm endosymbiotic bacteria, *Bacillus* sp., resulted in an inhibition zone of 16.88 mm for *Staphylococcus aureus* [54].

In our study, the EA-ME of *Planococcus* sp. and *K. aerogenes* inhibited the growth of *S. aureus* with inhibition zones of 17.0 and 15.0 mm, respectively. The EA-ME of *Bacillus amyloliquefaciens* isolated from a marine sponge showed strong antibacterial activity against *S. aureus* (inhibition zones 20.0 mm). In the same study, the EA-ME of *Alcaligenes faecalis* displayed high activity against *E. coli* (inhibition zone 16.0 mm) [56]. The EA-ME of *B. safensis* displayed moderate activity against *S. aureus* (MIC 1.25 mg/mL), and its extract had no impact on the remaining tested bacteria (Table 1). In the study of Sebola et al., the EA-ME of endophytic *B. safensis* extract showed activity against *E. coli* at a concentration of 0.25 mg/mL [5].

In another study, *B. safensis* isolated from *Ophioglossum reticulatum* displayed antibacterial activity against *S. aureus* and *E. coli*, with inhibition zones of 15.0 mm and 11.33 mm, respectively [57]. Such a difference in antibacterial activity could be attributed to the host, in which bacteria, in some instances, share the chemistry of their hosts to some extent [58]. In the study of Sebola and Mukherjee, *B. safensis* was isolated from ethnomedicinal plants in which bacteria may mimic the chemistry of their host plants and produce more bioactive and effective chemicals [58]. The association of bacteria with aphids was reported to perform several functions, such as providing essential amino acids, reproductive manipulations, and thermal adaptation [59]. In addition, chemicals produced by these symbiotic bacteria also help in defence against pathogenic microbes and predators [60,61,62,63].

For the antiproliferative activities, the data of the MTT assay showed the strong cytotoxicity of some EA-ME, including those of *K. aerogenes* (IC_50_: 67 ± 13.82 µg/mL for A549 cells and 38 ± 9.2 µg/mL for Hct116 cells) *P. agglomerans*, *E. avium* (IC_50_: 13 ± 14.13 µg/mL for A549 and 22 ± 11.13 µg/mL for Hct116 cells) and *Planococcus* sp. (25 ± 8.22 µg/mL for A549 cells and 48 ± 11.62 µg/mL for Hct116 cells). The EA-ME of endophytic *B. safensis* displayed notable cytotoxicity against A549 cells, with a 50% cell reduction at a concentration of 100 μg/mL [5]. In another study, the EA-ME of *B. safensis* isolated from sponges was reported to have cytotoxic activity against HepG2, HCT, and MCF7 [64]. However, in our study, the EA-ME of *B. safensis* showed weak or no cytotoxic activity against A549 and Hct116 cells at low concentrations (Figure 2A,B). Only at a high concentration of 100 µg/mL did the EA-ME result in 67.7% and 57.8 % cell reduction in A549 cells and Hct116, respectively. The difference in EA-ME activity across the studies could be attributed the host from which the bacteria was isolated [58]. Interestingly, the EA-ME of *Bacillus* sp. was able to reduce the cell viability of A549 cells to 0 % at 100 µg/mL [65]. The activity of *Bacillus* sp. against various types of cancer cells was reported [66,67,68,69,70]. These findings highlight the importance of *Bacillus* sp. as a source of biologically active compounds against cancer cells.

The chemical constituents of three EA-ME of *Planococcus* sp., *E. avium*, and *K. aerogenes* were analysed using GC-MS. Many of the identified compounds (Table 2, Table 3 and Table 4) were reported in several studies to have biological activities against pathogenic bacteria and cancer cells. For example, 2H-pyran, 2-(2,5-hexadiynyloxy) tetrahydro, identified in the three extracts, was isolated previously from *Aspergillus terreus* and reported to have antibacterial and antifungal activity [71]. It was also identified in *P. guajava*, *Pogostemon quadrifolius*, and many other medicinal plants with different biological activities [72,73,74]. In a recent study, the pyran compound was reported to act against *Salmonella enterica* serovar Typhi [75]. The compound dimethyl diphenyl tethylidyl pyrrolidine from *Planococcus* sp. was effective against several pathogenic bacteria such as *Porphyromonas gingivalis*, *S. aureus*, *S. pyrogenes* and *E. coli*, and reported to have antifungal activity against *Aspergillus niger*, *Candida albicans* and *Aspergillus clavatus* [76,77]. 3-Methyl-4-nitro-5-(1-pyrazolyl) pyrazole, which was identified in the EA-ME of *Planococcus* sp., was reported in the literature to have antimicrobial activities against *B. subtilis*, *S. aureus*, *P. fluorescens*, *P. aeruginosa* and *E. coli* [78]. In addition, it had anticancer activities against various types of cell lines [79,80,81].

Long-chain fatty acids such as oleic acid, cholest-5-en-3-ol and were also identified in one or more of the three EA-MEs. Oleic acid was reported to have antiproliferative activity against hepatocellular carcinoma cell lines, tongue squamous cell carcinomas and other cancer cells [82,83]. Ref. Dias, Raposo [84] suggested that this fatty acid has the potential to synergistically modify antibiotic activity. In addition, it had antibacterial activity against *S. aureus* and *E. coli* [85]. Cholest-5-en-3-ol was also reported to have antitumor and antibacterial activities against several pathogens, including *Acinetobacter baumannii* [86]. This biomarker is an identified sterol which has not been evaluated for its antitumor properties, but it has been reported to have anti-oxidative and antimicrobial properties [87]. Other compounds that were identified and reported to have major bioactive metabolites include di-(2-ethylhexyl) phthalate, isolated from River Nile-derived fungus *Aspergillus awamori* by Lotfy, Hassan [88], and pentadecanoic acid, which originates from some plants. Ruminant milk fat and fish oils have also been evaluated for their in vitro anticancer effects [89]. Therefore, some phenolic compounds, flavonoids and tannins in insects have bioactive characteristics and maintain stability during processing [90,91].

## 4. Materials and Methods

### 4.1. Aphid Sampling and Identification

Aphids were collected from different plant hosts including pomegranate, grapes and Taif roses. Aphid predators such as the ladybird *C. undecimpunctata*, lacewing larvae *C. carnea*, and ant *T. magnum* were collected from different sites located in Taif, Saudi Arabia. The samples were collected from May to September of 2021 (Table 5). For molecular identification of the collected insects, DNA extraction was accomplished using the QIAamp^®^ DNA Mini Kit (QIAGEN, Hilden, Germany) as described elsewhere [92]. The mitochondrial cytochrome oxidase gene (COI) was amplified with PCR, using the primer set (LCO1490) (F-5′-GGTCAACAAATCATAAAGATATTGG-3′) and (R HCO2) (R-5′-TAAACTTCAGGGTGACCAAAAAATCA-3′) [93]. The PCR reaction was performed as described in our previous study [92]. PCR products were visualized on 1.5% agarose gel using the BDA gel documentation system (Biometra, Göttingen, Germany). PCR products corresponding to the size of the amplified COI gene were retrieved from the gel, and DNA sequencing was performed on both strands using a 3130xl Genetic Analyzer (Biosystems; Thermo Fisher Scientific, Waltham, MA, USA). The raw sequence data were edited and assembled using Bio-edit software, version 7.2.5 (Ibis Biosciences, Carlsbad, CA, USA) and the Edit Seq program of the Lasergene software package, version 3.18 (DNA Star, Madison, WI, USA). The BLAST tool was used to identify the assembled sequences [94]. The assembled sequences were deposited in GenBank with the following accession numbers (aphids: MZ091377, MZ091379, and OL823183; aphid predators: ON149796, ON149797 and ant: ON149799).

### 4.2. Isolation of Insect Bacteria

Bacteria were isolated from the three identified aphid species, their two species of predators and one species of ant. The insects were surface-sterilized with 70% ethanol for 3 min and washed three times with sterile PBS to get rid of any surface contamination. The insects were then crushed and homogenized individually in nutrient broth media. Each isolation procedure was carried out in triplicate for each cultivar. Each triplicate suspension was diluted individually (10^−1^ through 10^−5^). Some 100 µL of each dilution was plated on nutrient agar plates. Plates were incubated in an inverted position for 2–3 days at 30 °C. Growing colonies were picked up, and then two rounds of purification were applied using nutrient agar plates. Purified bacterial isolates were picked, inoculated into 5 mL nutrient broth, and then incubated overnight at 37 °C. Following centrifugation, the pellets were subjected to the DNA extraction step.

### 4.3. DNA Extraction, Amplification and 16S rRNA Gene Sequencing

DNA was extracted individually from isolated bacteria using a DNeasy^®^ Blood and Tissue kit (QIAGEN) kit, following the manufacturer’s instructions. The V3 and V4 regions of the 16S rRNA gene was amplified using the following primer sets: Bakt_341F (CCTACGGGNGGCWGCAG), and Bakt_805R (GACTACHVGGGTATCTAATCC) [95]. The PCR reaction mixture contained 4 μL of FIREPol^®^ Ready to Load Master Mix (SolIS BioDyne, Tartu, Estonia), 0.6 μL of each primer, 2 μL of isolated DNA, and water to bring the total volume to 20 µL. The PCR reaction conditions were initial denaturation at 95 °C for 5 min, followed by 30 cycles of denaturation at 95 °C for 40 s, annealing at 55 °C for 2 min and extension at 72 °C for 1 min and a final extension step at 72 °C for 7 min. The resultant PCR products were analyzed on 1.5% agarose gels and visualized using the BDA gel documentation system (Biometra, Göttingen, Germany). PCR bands corresponding to the *16S rRNA* gene were excised from the gel and purified using Illustra GFX PCR DNA and a gel band purification kit (GE Healthcare). DNA sequencing was accomplished using 3130xl Genetic Analyzer (Biosystems; Thermo Fisher Scientific, Waltham, MA, USA). The raw sequence data were edited and assembled using Bioedit software, version 7.2.5 (Ibis Biosciences, Carlsbad, CA, USA), and the EditSeq program of the Lasergene software package, version 3.18 (DNAStar, Madison, WI, USA). Identification of bacterial isolates was achieved using the BLAST tool [94] and the *16S rRNA* gene sequences genes were deposited in GenBank with the following accession numbers (OP320676–OP320682, OQ351925–OQ351927), as shown in Table 5.

### 4.4. Preparation of Ethyl Acetate Fraction of Methanolic Extract (EA-ME) from Endosymbiotic Bacteria

Among the identified bacterial isolates, 10 out of 13 isolates (Table 5) were selected to investigate their antibacterial and anticancer activities. Each bacterium was inoculated into an Erlenmeyer flask containing 1 L of nutrient broth, and incubated in shaker incubator for 3–7 days at 200 rcf and 30 °C. After seven days of incubation, bacterial cultures were centrifuged, and sterile Amberlite^®^ XAD16 (60 g/L; Sigma, BCBR6696V) was added to the supernatant and shaken overnight at 200 rcf. The resin from each culture was collected individually after cheesecloth filtration into Erlenmeyer flasks; 300 mL of methanol was added to each flask and stirred for 2 h. The methanolic crude extract was completely dried using a rotary evaporator, weighted, and the obtained dry mass was further fractionated using 2 mL of ethyl acetate, based on the procedure reported in the previous studies [96,97,98,99] to yield the ethyl acetate fraction of methanolic extract (EA-ME). Some 5 µL of 1-bromodecane (4 mmol/L in ethyl acetate) was added, as an internal standard (IS), to each sample. The samples were stored at −80 °C.

### 4.5. Antibacterial Activities of EA-ME against Pathogenic Bacteria

The effect of each endosymbiont EA-ME was tested against five pathogenic bacteria, *Staphylococcus aureus* (ATCC6538), *Staphylococcus epidermidis* (ATCC14990), *Escherichia coli* (ATCC10536), *Klebsiella pneumoniae* (ATCC10031) and *Enterococcus cloacae* (ATCC13047), using the disk diffusion method [100]. The EA-ME were filtered through a Millipore filter. Sterile filter paper discs 8 mm in diameter were loaded with three different masses of each EA-ME: 2, 5, and 10 mg. The discs were allowed to dry at room temperature and placed over Mueller–Hinton Agar plates seeded with the pathogenic bacteria. Disks loaded with ampicillin and tetracycline were used as positive control at a concentration of 30 μg (Thermo Scientific, Applied Biosystems, Invitrogen, Gibco). The plates were incubated for 2 h at −4 °C to allow diffusion of the EA-ME, and then transferred to the incubator at 37 °C for 24 h. The inhibition zones around the discs were measured and were considered markers for antibacterial activity. 

### 4.6. Resazurin-Based 96-Well Plate Microdilution Assay for the Determination of MICs

The principle of this assay is based on the reduction of resazurin by living bacteria, resulting in colour changes from blue to pink and finally to colourless, due to oxygen deficiency in the medium. A slight colour change indicates the inability of bacteria to grow, and hence the antibacterial activity of the tested substance. Endosymbiont EA-ME that showed antibacterial activity were manipulated further to determine their minimum inhibitory concentration (MICs) and minimum bactericidal concentration (MBCs) using a microdilution assay. Serial dilutions from each EA-ME were prepared in MH broth, starting from 10 down to 0.039 mg/mL. The inoculum (100 μL) from each pathogenic bacterium was added to the assigned wells and the plate was incubated at 37 °C for overnight. On the next day, 10 μL of resazurin sodium salt dye solution (0.02% *w*/*v*) was added to the assigned wells and the plate was incubated for 2 h. After incubation, the plate was visually checked for colour change, and wells with a known concentration showing slight colour change were determined to be the MIC. The experiment was carried out in duplicate for each concentration of the EA-ME. The concentration above the MIC value was considered as that of MBC.

### 4.7. Antiproliferative Activity of the Endosymbiont EA m Extracts

The biological activity of the bacterial EA-ME against two types of cancer cell lines, adenocarcinoma human alveolar epithelial cells (A549), and human colon carcinoma (hct116), were tested in vitro using a 3-(4,5-Dimethylthiazol-2-yl)-2,5-Diphenyltetrazolium Bromide (MTT) colorimetric assay. The cells were maintained in Dulbecco’s Modified Eagle’s Medium (DMEM) supplemented with 10% fetal bovine serum, 2 mM L-glutamine, and 1% penicillin/streptomycin. The test was performed in 96-well plates, and the cells (5 × 10^4^ cells/mL) were seeded into the assigned wells and were incubated overnight at 37 °C, 5% CO_2_, and 99% humidity. Two-fold serial dilutions of the EA-ME, starting from 100 μg/mL down to 0.39 μg/mL, were prepared in DMEM and added to the assigned wells. Control wells received ethyl acetate only and the plates were incubated for four days. The MTT was added to the cells, and the plates were incubated under growth conditions for two hours. The medium was then removed, and the cells were washed two times in phosphate-buffered saline (PBS). The formed formazan crystals were dissolved in Isopropanol. The cytotoxic activity of the anticancer drug dasatinib against the two cell lines A549 and Hct116 was used as a positive control for comparative purposes. The compound was diluted to the same concentrations of the tested extracts. The experiment was performed in triplicate for each concentration, the absorbance was measured at 595 nm and the average values obtained were considered. The following formula was used to calculate cell viability (Equation (1)) [101]:

(1) $$\%\;cell\;viability=\frac{A1-A0}{Ac-A0}\times 100$$

where *A*0, *Ac* and *A*1 are, respectively, the absorbance of blank, control solution and the EA-ME at 595 nm. The *IC*_50_ % cell viability values were determined from % cell viability and the concentration curve, according to the following Equation (2) [102]:

(2) $${IC}_{50}=\frac{a-c}{b-c}\times 100$$

where *a*, *b* and *c* are, respectively, the absorbance at each concentration of the anticancer reagent, the absorbance at 0 µM of the anticancer reagent, and the absorbance of the blank.

### 4.8. Gas Chromatography–Mass Spectroscopy Analysis (GC-MS)

Samples were analysed sing gas chromatography–mass spectrometer GC-MS TSQ (Thermo Scientific, Austin, TX, USA) with a direct capillary column TG–5MS (30 m × 0.25 mm × 0.25 µm film thickness). The run was performed according to the protocol of Abd El-Kareem, Rabbih (2016) [103]. The column oven temperature was initially held at 50 °C and then increased by 5 °C/min to 250 °C hold for 2 min, and then increased to a final temperature of 300 °C by 30 °C/min, and hold for 2 min. The injector and MS transfer line temperatures were kept at 270 °C. Helium was used as a carrier gas at a constant flow rate of 1 mL/min. Some 1 µL of samples was injected automatically using an autosampler AS1300 coupled with GC in the spitless mode. The mass spectrometry conditions were as follows: the electron ionization source was set at 70 eV, the MS source temperature at 200 °C, and the solvent cut time was 4 min. The MS transfer line temperatures were kept at 260 °C. The mass spectrometer was run in full scan mode (*m*/*z* 50–650). 

The components were identified using comparison of their mass spectra with those of Wiley09 and the National Institute of Standard Technology (NIST 14) mass spectral database. The strategy of accepting the identification of compounds based on the reverse match for untargeted analysis was adopted from Alsufyani et. al., (2021) [104]. The retention time of compounds was adjusted to that of IS (Rt ≈ 23:09–23:13 min). 

### 4.9. Statistical Analysis

Antibacterial and anticancer results were statistically analysed using Microsoft Excel. The measurements of inhibition zones, MIC values, and IC_50_ values were calculated and expressed as means ± of standard deviations of the triplicates. The IC_50_ values of the tested extracts and the positive control, dasatinib, against the two cell lines were compared with the control cells using an unpaired Student’s *t*-test to calculate the statistical significance. *p* values of ≤0.05 were considered as statistically significant.

## 5. Conclusions

In conclusion, this study highlights the importance of aphid endosymbiotic bacteria, predators and ants as sources of bioactive compounds for cancer and bacterial pathogens. Among the bacteria tested, the EA-EM from *Planococcus* sp., *K.* *aerogenes*, and *E. avium* were the most effective against pathogenic bacteria and cancerous cell lines. Large-scale studies to investigate more endosymbiotic bacteria of aphids and other insects may lead to the discovery of new bacterial species with potent anticancer and antimicrobial activities.

## Figures and Tables

**Table 1:** Antibacterial activities of the EA-ME of the endosymbiont at 10 mg/mL concentration, and MIC values.

EA-ME		Pathogenic Bacteria				
		*S. aureus*	*S. epidermidis*	*E. cloacae*	*K. pneumoniae*	*E. coli*
*Planococcus* sp.	Inhibition zone	17.00 ± 0.75	16.00 ± 0.91	-	11.00 ± 0.35	-
	MIC	0.3125	0.3125		0.625	
	MBC	0.3125	0.3125		1.25	
*Enterobacter* sp.		-	-	-	-	-
*S. odorifera*		-	-	-	-	-
*K. aerogenes*	Inhibition zone	15.00 ± 0.41	16.00 ± 0.29	-	20.00 ± 1.11	18.00 ± 0.65
	MIC	0.625	0.3125		0.156	0.156
	MBC	0.625	0.3125		0.625	0.3125
*S. fonticola*		-	-	-	-	-
*P. stuartii*	Inhibition zone	09.00 ± 0.22	-	-	-	-
	MIC	1.25				
	MBC	2.5				
*E. avium*	Inhibition zone	10.00 ± 0.13	17.00 ± 0.37	11.00 ± 0.39	-	-
	MIC	1.25	0.3125	0.625		
	MBC	2.5	0.625	1.25		
*P. agglomerans*		-	-	-	-	-
*B. megaterium*		-	-	-	-	-
*B. safensis*	Inhibition zone	10.00 ± 0.45	-	-	-	-
	MIC	1.25				
	MBC	2.5				
Ampicillin		19.00 ± 0.52 0.3125	16.00 ± 0.95 0.625	15.00 ± 0.37 0.625	-	11.00 ± 0.33 0.625
Tetracycline		35.00 ± 0.54 0.039	33.00 ± 0.36 0.039	25.00 ± 0.22 0.078	29.00 ± 0.63 0.078	30.00 ± 0.54 0.039

Data presented in the table are means of three replicates SD (*n* = 3). MIC/MBC values are measured as mg/mL.

**Table 2:** Chemical compositions of *Planococcu.* sp. ethyl acetate fraction of methanol extract.

Compound Name	RT	Molecular Formula	Molecular Weight
2-(2,5-Hexadiynyloxy) tetrahydro-2H-pyran ??	4.10	C_11_H_14_O_2_	178
Dimethyl diphenyl tethylidyl pyrrolidine ??	4.97	C_20_H_23_N	277
2-Aminoethanethiol hydrogen sulfate ester ??	6.27	C_2_H_7_NO_3_S_2_	157
3-Methyl-4-nitro-5-(1-pyrazolyl) pyrazole ??	6.48	C_7_H_7_N_5_O_2_	193
4a,5,8,8a-Tetrahydro-4,4a-dimethyl-2(1H)-naphthalenone ??	8.00	C_12_H_16_O	176
Folinic acid ??	8.15	C_20_H_23_N_7_O_7_	473
2,3-Dihydro-2-thioxo-3-diallylaminom ethyl benzoxazol ??	9.14	C_14_H_16_N_2_OS	260
1-(á-d-Arabinofuranosyl)-4-O-difluoro methyl uracil ???	14.79	C_10_H_12_F_2_N_2_O_6_	294
4-(1,1-Dimethylethoxy) benzoic acid ??	15.05	C_11_H_14_O_3_	194
Methyl 13,16-octadecadiynate ?	19.09	C_19_H_30_O_2_	290
(2-Phenyl-1,3-dioxolan-4-yl)methyl 9-octadecenoate ???	20.96	C_28_H_44_O_4_	444
Leukotriene D4 methyl ester ??	22.65	C_26_H_42_N_2_O_6_S	510
Z-8-Methyl-9-tetradecenoic acid ??	22.72	C_15_H_28_O_2_	240
9,10-Dihydroxy methyl octadecanoat??	23.38	C_19_H_38_O_4_	330
2,3-Bis(acetyloxy)propyl dodecanoic acid,??	23.90	C_19_H_34_O_6_	358
Estra-1,3,5(10)-trien-17á-ol ??	24.80	C_18_H_24_O	256
D-Fructose, diethyl mercaptal, pentaacetate ??	25.17	C_20_H_32_O_10_S_2_	496
7,8,15,16-Tetramethyl-1,9-dio xacyclohexadeca-4,13-diene-2,10-dione ???	25.27	C_18_H_28_O_4_	308
D-Fructose, diethyl mercaptal, pentaacetate	25.58	C_20_H_32_O_10_S_2_	496
(z,z,z)-6,9,12-Phenylmethyl octadecatrienoate, ester,?	39.05	C_25_H_36_O_2_	368
Cholest-5-en-3-ol (3á) ?	39.57	C_27_H_46_O	386

Structures with a reverse match between 800 and 700 were tagged with a “?”, structures with a reverse match between 700–600 with “??”, and structures with a reverse match below 600 with “???”. Reverse match factors below 500 was not accepted.

**Table 3:** Chemical compositions for *E. avium* ethyl acetate fraction of methanol extract.

Compound Name	RT	Molecular Formula	Molecular Weight
5.Alpha pregnane-3.alpha.,11.beta.,20.beta.,21-tetrol, cyclic 20,21-(2-methyl-2-propaneboronate) ??	4.09	C_25_H_43_BO_4_	418
2-Octyl methyl cyclopropanedodecanoiate ??	14.77	C_24_H_46_O_2_	366
Methyl-16-hydroxy-hexadecanoate ??	19.06	C_17_H_34_O_3_	286
Methyl hexandecanoiate ?	22.11	C_17_H_34_O_2_	270
N-(3-Chlorobenzylidene)-10-undecenoic acid hydrazide ???	23.40	C_18_H_25_ClN_2_O	320
Pyrrolo[1,2-a]pyrazine-1,4-dione, hexahydro-3-(2-methylpropyl) ??	24.85	C_11_H_18_N_2_O_2_	210
Pentadecanoic acid ??	24.95	C_15_H_30_O_2_	242
13-Methyl oxacyclotetradecan-2-one ??	26.00	C_14_H_26_O_2_	226
Methyl-8,11-octadecadiynoic acid ??	27.14	C_19_H_30_O_2_	290
1,2-Benzene dicarboxylic acid ?	33.57	C_24_H_38_O_4_	390
Mono(2-ethylhexyl) phthalate ??	36.42	C_16_H_22_O_4_	278
Arachidonic acid ??	39.41	C_20_H_32_O_2_	304
Cholest-5-en-3-yl (9z)-9-octadecenoate ?	39.57	C_45_H_78_O_2_	650

Structures with a reverse match between 800 and 700 were tagged with a “?”, structures with a reverse match between 700–600 with “??”, and structures with a reverse match below 600 with “???”. Reverse match factors below 500 was not accepted.

**Table 4:** Chemical compositions for *K. aerogenes* ethyl acetate fraction of methanol extract.

Compound Name	RT	Molecular Formula	Molecular Weight
2-(2,5-Hexadiynyloxy) tetrahydro-2H-pyran ??	4.09	C_11_H_14_O_2_	178
N-(p-methoxybenzyl) acetamide ??	4.62	C_10_H_13_NO_2_	179
1-(1-Cyclopenten-1-yl)- pyrrolidine ?	14.74	C_12_H_16_S	192
Methyl 13,16-octadecadiynoiate?	19.07	C_19_H_30_O_2_	290
2,4-Dimethyl hexanedioic acid ??	23.42	C_8_H_14_O_4_	174
Oleic acid ?	24.87	C_18_H_34_O_2_	282
(Z)-9-Methyl octadecenoate?	26.30	C_19_H_36_O_2_	296
Methyl cyclopentane undecanoiate ?	26.81	C_17_H_32_O_2_	268
Didodecyl phthalate ?	33.58	C_32_H_54_O_4_	502
2-Aminoethanethiol hydrogen sulfate ?	39.57	C_2_H_7_NO_3_S_2_	157

Structures with a reverse match between 800 and 700 were tagged with a “?”, and structures with a reverse match between 700–600 with “??”. Reverse match factors below 500 was not accepted.

**Table 5:** Insect samples, collection dates and collection sites in the Taif governorate.

Insects	Insects’ Species	Collection Date	Endosymbiotic Bacteria	Accession Numbers
Pomegranate aphid	*A. punicae*	27 June 2021	*Planococcus* sp.	OP320679
			*Bacillus megaterium*	OQ351927
Taif rose aphid	*M. rosae*	11 September 2021	*Pantoea agglomerans*	OP320678
			*Bacillus safensis*	OQ351925
Grape aphid	*A. illinoisensis*	21 May 2021	*Bacillus* sp.	-
Lacewing	*C. carnea*	27 June 2021	*Klebsiella aerogene*	OP320677
			*Serratia fonticola*	OP320681
			*Providencia stuartii*	OP320680
Eleven-spot ladybird	*C. undecimpunctata*	27 June 2021	*Enterobacter* sp.	OP320676
			*Bacillus* sp.	-
			*Serratia odorifera*	OP320682
Ant	*T. magnum*	27 June 2021	*Bacillus* sp.	-
			*Serratia odorifera*	OP320682

## Data Availability

The datasets generated during the current study are available in the [National Center for Biotechnology Information: ncbi] repository, [https://www.ncbi.nlm.nih.gov/nucleotide/ accessed on 5 April 2022]. The GenBank accession numbers of the data are shown in Table A1 of Appendix A.

## Supplementary Materials

The following supporting information can be downloaded at: https://www.mdpi.com/article/10.3390/molecules28104255/s1, Figure S1: GC-MS chromatograms of bioactive compounds extracted from the ethyl acetate fraction of the following bacteria (A) *Planococcus* sp., (B) *K. aerogenes*, and (C) *E. avium*. 1-bromodecane is IS (RT = 23.09–23.13); Table S1: Other chemical compounds detected by GC/MS of EA-ME of *E. avium* and *K. aerogene* bacteria isolated respectively from ant *A. punicae* and Lacewing (*C. carnea*). References [36,38,39,88,105,106,107,108,109,110,111] are cited in the Supplementary Materials.

## Appendix A

**Table A1:** GenBank accession numbers of the data.

No	Species	Sequence	Accession Number
Aphid and aphid predators and ants sequences of Cytochrome oxidase subunit I			
1	*Aphis illinoisensis*	GGATCTTCACTTAGAATTTTAATTCGATTAGAATTAAGTCAAATTAATTCAATTATTAACAATAATCAATTATATAATGTAATCGTAACAATTCATGCTTTTATTATAATTTTTTTTATAACTATACCTATTGTAATTGGAGGATTTGGAAATTGGTTAATTCCTATAATAATAGGATGTCCAGATATATCTTTCCCACGACTAAATAATATTAGATTTTGGTTACTACCACCATCATTAATAATAATAATTTGTAGATTTATAATTAATAATGGAACAGGAACAGGATGGGCTATTTATCCACCTTTATCAAATAATATTGCTCACAATAATATTTCAGTTGATCTAACTATTTTTTCTCTTCATATAGCAGGAATTTCATCAATTTTAGGAGCAATTAATTTTATTTGTCAATTTTAAACATAATACCACATAATATAAAACTAAATCAAATTCCTTTATTCCCATGGTCAATCTTAATTACAGCCATATTATTAATTTTATCTTTACCAGTTTTAGCTGGTGCTATTACAATATTATTAACTGATCGAAATTTAAATACATCATTTTTTGATCCAGCAGGAGGAGGAGACCCTATTCTTTATCAACATTTATTCTGGTTTTTT	MZ091377
2	*Aphis punicae*	GGTTCTTCTCTTAGAATTTTAATCCGATTAGAATTAAGTCAAATTAATTCAATTATTAATAATAATCAACTATATAATGTAATTGTTACAATTCATGCTTTTATTATAATTTTTTTTATAACTATACCAATCGTTATTGGAGGTTTTGGAAATTGGTTAATTCCTATAATAATAGGATGCCCAGATATATCTTTCCCACGACTAAATAATATTAGATTCTGGTTATTACCACCCTCATTAATAATAATAATTTGCAGATTTATAATTAATAATGGAACAGGAACAGGATGGACTATTTATCCACCTTTATCAAATAACATTGCTCATAATAATATTTCAGTAGACTTAACTATTTTTTCTTTACATTTAGCAGGTATTTCATCAATTTTAGGAGCAATTAATTTCATCTGCACTATCTTAAATATAATACCCAATAATATAAAATTAAATCAAATTCCTTTATTTCCATGGTCAATTTTAATTACAGCTATATTATTAATTTTATCCTTACCCGTATTAGCTGGTGCTATTACTATATTATTAACAGATCGAAATTTAAATACATCATTTTTTGATCCAGCAGGTGGTGGAGACCCTATTCTTTATCAACATTTATTTTGGTTTTTT	MZ091379
3	*Macrosiphum rosae*	ACTCTTAGAATTTTAATTCGATTAGAATTAAGACAAATTAATTCTATTATTAATAATAATCAATTATATAATGTAATTGTTACAATTCATGCTTTTATTATAATTTTTTTTATAACTATACCAATTGTAATTGGAGGATTTGGAAATTGGTTAATTCCTATAATAATAGGATGCCCTGATATATCATTTCCACGTTTAAATAATATTAGATTTTGGTTATTACCTCCATCATTAATAATAATAATTTGTAGATTTTTAATTAATAACGGTACAGGAACAGGATGGACAATTTATCCACCTTTATCAAACAATATTGCACACAATAATATTTCAGTTGATTTAACTATTTTTTCTCTGCATTTAGCAGGAATTTCATCAATCTTAGGAGCAATTAACTTTATTTGTACAATTCTTAATATAATACCAAATAATTTAAAACTTAATCAAATTCCTCTCTTTCCTTGGTCAATTTTAATTACAGCTATTTTACTAATTTTATCTTTACCAGTTTTAGCCGGTGCTATTACAATATTACTAACTGATCGTAATTTAAATACATCATTTTTTGATCCAGCAGGAGGAGGAGACCCTATTTTATATCAACATTTATTTTGGTTTTTG	OL823183
4	*Chrysoperla carnea*	GGATCATCTCTAAGTTTATTGATTCGAGCTGAATTAGGTCAACCAGGTTCTTTAATTGGTGATGATCAAATTTATAATGTAATTGTTACAGCACATGCTTTTATTATAATTTTTTTTATAGTAATACCTATTGTAATTGGAGGTTTTGGTAATTGGTTAGTTCCTTTAATATTAGCTGCTCCTGATATAGCTTTTCCACGAATAAATAATATAAGTTTCTGGATATTACCTCCTTCATTAACTTTATTACTTGCTTCTTCTATAGTAGAAAGAGGAGCTGGAACTGGTTGGACAGTTTACCCTCCTTTATCATCAAGAATTGCTCATGCTGGAGCTTCTGTTGATTTAGCTATTTTTAGTTTACATCTTGCCGGTATTTCATCAATTTTAGGAGCAGTAAATTTTATTACAACAGTAATTAATATACGATTAAGTTATATAACATTAGATCGTATACCATTATTTGTATGGTCAGTAGTAATTACAGCTTTATTATTATTACTTTCATTACCTGTATTAGCTGGTGCTATTACTATATTATTAACTGATCGTAATTTAAATACTTCATTTTTTGA	ON149796
5	*Coccinella undecimpunctata*	GGATCATCTCTAAGAATCTTAATTCGGCTAGAACTTGGTACTACAAACAGATTAATTGGAAATGACCAAATTTATAATGTTATTGTAACAGCTCATGCATTTATTATAATTTTTTTCATAGTAATACCAATTATAATTGGAGGATTTGGAAATTGGTTAGTTCCCCTAATAATTGGGGCACCTGATATAGCTTTCCCACGTTTAAATAATATAAGATTCTGGTTATTACCTCCTGCATTAACTCTCTTAATCATTAGAAGATTAGTAGAAATAGGGGCAGGAACAGGTTGGACTGTTTACCCACCTTTATCTTCTAATTTAGCTCATAATGGGCCTTCTGTAGATTTAGTAATTTTTAGATTACACTTAGCAGGAATTTCTTCAATTCTTGGAGCTGTAAATTTCATCTCTACAATTATAAATATACGCCCCTTTGGAATAAATTTGGATAAAACTCCTTTATTTGTTTGGTCAGTACTTATTACTGCTATTTTATTACTTCTTTCATTACCAGTTTTAGCTGGGGCTATTACAATACTATTAACTGACCGTAATATTAATACATCTTTTTTTGA	ON149797
6	*Tapinoma magnum*	GGATCATCTCTAAGAATAATTATCCGTATTGAATTAGGAACATGTGGAGCATTAATTAATAATGATCAAATTTATAATTCAATTGTTACAGGACATGCTTTTATTATAATTTTTTTTATAGTTATACCTTTTATAATTGGTGGATTTGGAAATTTTTTAGTCCCATTAATATTAGGTGCACCAGATATGGCTTATCCTCGAATAAATAATATAAGATTTTGGTTATTACCCCCATCAATTTTATTATTAACTATTAGAAATTTTATCAGATCAGGGGTAGGTACTGGTTGGACAGTATACCCACCCTTAGCATCTAATATTTATCATAACGGACCTTCAGTAGATTTAGCTATTTTTTCTTTACATATTGCAGGAATATCATCAATCTTAGGCGCAATTAATTTTATTTCTACAATTATTAATATACATCATAAAAATTTTTCTATTGATAAAATTCCTTTATTAGTATGGTCAATTTTAATTACTGCAATTTTATTACTTTTATCTCTTCCAGTTTTAGCAGGAGCAATTACTATGTTATTAACTGATCGAAATTTAAATACATCATTTTTTGA	ON149799
**16s rRNA sequences of Bacterial isolates**			
7	*Enterobacter* sp.	ATTGACGTTACCCGCAGAAGAAGCACCGGCTAACTCCGTGCCAGCAGCCGCGGTAATACGGAGGGTGCAAGCGTTAATCGGAATTACTGGGCGTAAAGCGCACGCAGGCGGTCTGTCAAGTCGGATGTGAAATCCCCGGGCTCAACCTGGGAACTGCATTCGAAACTGGCAGGCTAGAGTCTTGTAGAGGGGGGTAGAATTCCAGGTGTAGCGGTGAAATGCGTAGAGATCTGGAGGAATACCGGTGGCGAAGGCGGCCCCCTGGACAAAGACTGACGCTCAGGTGCGAAAGCGTGGGGAGCAAACAGGATTAGATACCCCGGTAGTCA	OP320676
8	*Klebsiella aerogenes*	ATTGACGTTACCCGCAGAAGAAGCACCGGCTAACTCCGTGCCAGCAGCCGCGGTAATACGGAGGGTGCAAGCGTTAATCGGAATTACTGGGCGTAAAGCGCACGCAGGCGGTCTGTCAAGTCGGATGTGAAATCCCCGGGCTCAACCTGGGAACTGCATTCGAAACTGGCAGGCTAGAGTCTTGTAGAGGGGGGTAGAATTCCAGGTGTAGCGGTGAAATGCGTAGAGATCTGGAGGAATACCGGTGGCGAAGGCGGCCCCCTGGACAAAGACTGACGCTCAGGTGCGAAAGCGTGGGGAGCAAACAGGATTAGATACCCCGGTAGTCAA	OP320677
9	*Pantoea agglomerans*	ATTGACGTTACCCGCAGAAGAAGCACCGGCTAACTCCGTGCCAGCAGCCGCGGTAATACGGAGGGTGCAAGCGTTAATCGGAATTACTGGGCGTAAAGCGCACGCAGGCGGTTTGTTAAGTCTGATGTGAAATCCCCGGGCTCAACCGGGGAACTGCATTGGAAACTGGGAGGCTTGAGTCTTGTAGAGGGGGGTAGAATTCCAGGTGTAGCGGTGAAATGCGTAGAGATCTGGAGGAATACCGGTGGCGAAGGCGGCCCCCTGGACAGAGACTGACGCTCAGGTGCGAAAGCGTGGGGAGCAAACAGGATTAGATACCCCCGGTAGTCAA	OP320678
10	*Planococcus* sp.	CTTGACGGTACCTCACCAGAAAGCCACGGCTAACTACGTGCCAGCAGCCGCGGTAATACGTAGGTGGCAAGCGTTGTCCGGAATTATTGGGCGTAAAGCGCGCGCAGGCGGTTCCTTAAGTCTGATGTGAAAGCCCACGGCTCAACCGTGGAGGGTCATTGGAAACTGGGGAACTTGAGTGCAGAAGAGGAAAGTGGAATTCCACGTGTAGCGGTGAAATGCGTAGAGATGTGGAGGAACACCAGTGGCGAAGGCGACTTTCTGGTCTGTAACTGACGCTGAGGCGCGAAAGCGTGGGGAGCAAACAGGATTAGATACCCCGGTAGTCA	OP320679
11	*Providencia stuartii*	ATTGACGTTACCGACAGAAGAAGCACCGGCTAACTCCGTGCCAGCAGCCGCGGTAATACGGAGGGTGCAAGCGTTAATCGGAATTACTGGGCGTAAAGCGCACGCAGGCGGTTGATTAAGTTAGATGTGAAATCCCCGGGCTTAACCTGGGAATGGCATCTAAAACTGGTCAGCTAGAGTCTTGTAGAGGGGGGTAGAATTCCATGTGTAGCGGTGAAATGCGTAGAGATGTGGAGGAATACCGGTGGCGAAGGCGGCCCCCTGGACAAAGACTGACGCTCAGGTGCGAAAGCGTGGGGAGCAAACAGGATTAGATACCCCGGTAGTCAA	OP320680
12	*Serratia fonticola*	ATTGACGTTACTCGCAGAAGAAGCACCGGCTAACTCCGTGCCAGCAGCCGCGGTAATACGGAGGGTGCAAGCGTTAATCGGAATTACTGGGCGTAAAGCGCACGCAGGCGGTTTGTTAAGTCAGATGTGAAATCCCCGAGCTTAACTTGGGAACTGCATTTGAAACTGGCAAGCTAGAGTCTTGTAGAGGGGGGTAGAATTCCAGGTGTAGCGGTGAAATGCGTAGAGATCTGGAGGAATACCGGTGGCGAAGGCGGCCCCCTGGACAAAGACTGACGCTCAGGTGCGAAAGCGTGGGGAGCAAACAGGATTAGATACCCCGGTAGTCA	OP320681
13	*Serratia odorifera*	ATTGACGTTACTCGCAGAAGAAGCACCGGCTAACTCCGTGCCAGCAGCCGCGGTAATACGGAGGGTGCAAGCGTTAATCGGAATTACTGGGCGTAAAGCGCACGCAGGCGGTTTGTTAAGTCAGATGTGAAATCCCCGCGCTTAACGTGGGAACTGCATTTGAAACTGGCAAGCTAGAGTCTCGTAGAGGGGGGTAGAATTCCAGGTGTAGCGGTGAAATGCGTAGAGATCTGGAGGAATACCGGTGGCGAAGGCGGCCCCCTGGACGAAGACTGACGCTCAGGTGCGAAAGCGTGGGGAGCAAACAGGATTAGATACCCCGGTAGTCAAGCG	OP320682
14	*Bacillus safensis*	CTTGACGGTACCTAACCAGAAAGCCACGGCTAACTACGTGCCAGCAGCCGCGGTAATACGTAGGTGGCAAGCGTTGTCCGGAATTATTGGGCGTAAAGGGCTCGCAGGCGGTTTCTTAAGTCTGATGTGAAAGCCCCCGGCTCAACCGGGGAGGGTCATTGGAAACTGGGAAACTTGAGTGCAGAATAGGAGAGTGGAATTCCACGTGTAGCGGTGAAATGCGTAGAGATGTGGAGGAACACCAGTGGCGAAGGCGACTCTCTGGTCTGTAACTGACGCTGAGGAGCGAAAGCGTGGGGAGCGAACAGGATTAGATACCCTGGTAGTCC	OQ351925
15	*Enterococcus avium*	CTTGACGGTATCTAACCAGAAAGCCACGGCTAACTACGTGCCAGCAGCCGCGGTAATACGTAGGTGGCAAGCGTTGTCCGGATTTATTGGGCGTAAAGCGAGCGCAGGCGGTTTCTTAAGTCTGATGTGAAAGCCCCCGGCTCAACCGGGGAGGGTCATTGGAAACTGGGAAACTTGAGTGCAGAAGAGGAGAGTGGAATTCCATGTGTAGCGGTGAAATGCGTAGATATATGGAGGAACACCAGTGGCGAAGGCGGCTCTCTGGTCTGTAACTGACGCTGAGGCTCGAAAGCGTGGGAGCAAACAGGATTAGATACCCTGGTAGTCC	OQ351926
16	*Bacillus megaterium*	CTTGACGGTACCTAACCAGAAAGCCACGGCTAACTACGTGCCAGCAGCCGCGGTAATACGTAGGTGGCAAGCGTTATCCGGAATTATTGGGCGTAAAGCGCGCGCAGGCGGTTTCTTAAGTCTGATGTGAAAGCCCACGGCTCAACCGTGGAGGGTCATTGGAAACTGGGGAACTTGAGTGCAGAAGAGAAAAGCGGAATTCCACGTGTAGCGGTGAAATGCGTAGAGATGTGGAGGAACACCAGTGGCGAAGGCGGCTTTTTGGTCTGTAACTGACGCTGAGGCGCGAAAGCGTGGGGAGCAAACAGGATTAGATACCCTGGTAGTCC	OQ351927
