# Supplementary material for: GC Analysis, Anticancer, and Antibacterial Activities of Secondary Bioactive Compounds from Endosymbiotic Bacteria of Pomegranate Aphid and Its Predator and Protector
Source: Molecules. 2023 May 22;28(10):4255. doi: 10.3390/molecules28104255 (PMC10222457; doi:10.3390/molecules28104255)
Supplement: Supplementary file 1 [file molecules-28-04255-s001.zip › molecules-2335544-supplementary.pdf]

GC Analysis, Anticancer, and Antibacterial Activities of Secondary Bioactive Compounds from Endosymbiotic Bacteria of Pomegranate Aphid and Its Predator and Protector

Taghreed Alsufyani [1,2,*], Najwa Al-Otaibi [2,3], Noura J. Alotaibi [2,3], Nour Houda M'sakni [1,2,4] and Eman M. Alghamdi [5]

[1] Department of Chemistry, College of Science, Taif University, P.O. Box 11099, Taif 21944, Saudi Arabia; nour.h@tu.edu.sa
[2] High Altitude Research Centre, Taif University, P.O. Box 11099, Taif 21944, Saudi Arabia; naotaibi@tu.edu.sa (N.A.-O.); n.alotaibi@tu.edu.sa (N.J.A.)
[3] Department of Biology, College of Science, Taif University, P.O. Box 11099, Taif 21944, Saudi Arabia
[4] Laboratory of the Interfaces and Advanced Materials (LIMA), Science Faculty, Monastir University, P.O. Box 05019, Monastir 5019, Tunisia
[5] Chemistry Department, Faculty of Science, King Abdul Aziz University, P.O. Box 80200, Jeddah 21589, Saudi Arabia; emalghamdi@kau.edu.sa
* Correspondence: taghreed.alsufyani@tu.edu.sa

Supplementary Figures

3 GC/MS figures

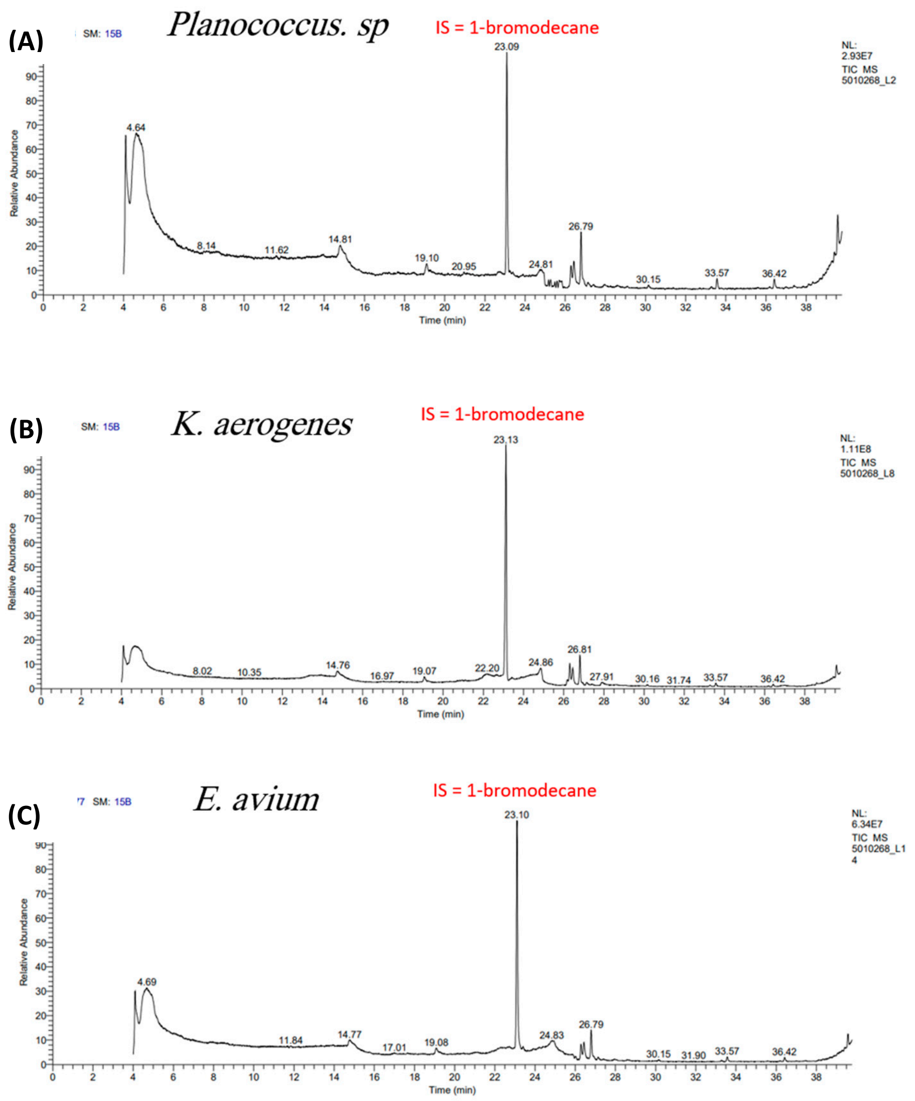

Figure S1: GC-MS chromatograms of bioactive compounds extracted from the ethyl acetate fraction of the following bacteria (A) *Planococcus* sp., (B) *K. aerogenes*, and (c) *E. avium*. 1-bromodecane is IS (RT = 23.09 – 23.13).

Supplementary Table

Table S1: Other chemical compounds detected by GC/MS of EA-ME of *E. avium* and *K. aerogene* bacteria isolated respectively from ant *A. punicae* and Lacewing (*C. carnea)*

Compounds	Origin	Biologic activities	References
Di-(2-ethylhexyl) Phthalate (DEHP)	Synthetic materials<br>+<br>Bio-organisms, such as microbes, algae, plants	Plasticizer<br>+<br>anti-bacterial, larvicidal and cytotoxic<br>+<br>allelopathic, antimicrobial, insecticidal to improve the competitiveness of plants, and microorganisms to better adapt to biotic and abiotic stress.	[1-4]
Mono(2-ethylhexyl) phthalate (MEHP)	Synthetic materials<br>+<br>Secondary metabolites of DEHP	Plasticizer	[5, 6]
Didodecyl phthalate (DDDP)	Synthetic materials<br>+<br>in the Environment	In silico drug targeting agent<br>+<br>Phthalate Plasticizers	[7]
Pregnane- 3,11,20,21-tetrol, cyclic 20,21-(butyl boronate)	Host plant<br>n-hexane fraction of *Phlomis stewartia*<br><br>*methanolic extract of* Epilobium angustifolium L.	Hormonal Compounds	[8]<br><br>[9]
Acetamide, n-(p-methoxybenzyl)	Synthetic compound	antimicrobial, anticancer, antioxidant, insecticidal against *cowpea aphid and Aphis craccivora* Koch (Homoptera: Aphididae)	[10, 11]

References

1. Rajamanikyam, M., et al., *Isolation and characterization of phthalates from Brevibacterium mcbrellneri that cause cytotoxicity and cell cycle arrest.* Excli j, 2017. **16**: p. 375-387.
2. Lotfy, M.M., et al., *Di-(2-ethylhexyl) Phthalate, a major bioactive metabolite with antimicrobial and cytotoxic activity isolated from River Nile derived fungus Aspergillus awamori.* Beni-Suef University Journal of Basic and Applied Sciences, 2018. **7**(3): p. 263-269.
3. Huang, L., et al., *Phthalic Acid Esters: Natural Sources and Biological Activities.* Toxins (Basel), 2021. **13**(7).
4. Habib, M.R. and M.R. Karim, *Antimicrobial and Cytotoxic Activity of Di-(2-ethylhexyl) Phthalate and Anhydrosophoradiol-3-acetate Isolated from Calotropis gigantea (Linn.) Flower.* Mycobiology, 2009. **37**(1): p. 31-6.
5. Koch, H.M., R. Preuss, and J. Angerer, *Di(2-ethylhexyl)phthalate (DEHP): human metabolism and internal exposure – an update and latest results1.* International Journal of Andrology, 2006. **29**(1): p. 155-165.
6. Lan, K.-C., et al., *Plasticizer Di-(2-ethylhexyl) Phthalate and Its Metabolite Mono(2-ethylhexyl) Phthalate Inhibit Myogenesis in Differentiating Mouse and Human Skeletal Muscle Cell Models.* Applied Sciences, 2022. **12**(18): p. 9195.
7. Jananie, R.K., V. Priya, and K. Vijayalakshmi, *Secondary metabolites of Cynodon dactylon as an antagonist to angiotensin II type1 receptor: Novel in silico drug targeting approach for diabetic retinopathy.* J Pharmacol Pharmacother, 2012. **3**(1): p. 20-5.
8. Farooq, A., et al., *Evaluation of Antioxidant, Antimicrobial activity and GC-MS analysis of Phlomis stewartii.* Pure and Applied Biology, 2019. **8**: p. 2420-2433.
9. Sayik, A., et al., *DNA- Binding, Biological Activities and Chemical Composition of Wild Growing Epilobium angustifolium L. Extracts from Canakkale, Turkey.* Journal of the Turkish Chemical Society, Section A: Chemistry, 2017. **4**: p. 811-840.
10. Abdussalam-Mohammed, W., et al., *Biofabrication of Silver Nanoparticles Using Teucrium Apollinis Extract: Characterization, Stability, and Their Antibacterial Activities.* Chemistry, 2023. **5**(1): p. 54-64.
11. El-Dean, A., et al., *Synthesis and biological activity of 2-((3-Cyano-4,6-distyrylpyridin-2- yl) thio) acetamide and its cyclized form.* 2020. **1**: p. 46-50.
